# The Clock Takes Shape—24 h Dynamics in Genome Topology

**DOI:** 10.3389/fcell.2021.799971

**Published:** 2022-01-03

**Authors:** Kévin Tartour, Kiran Padmanabhan

**Affiliations:** Institut de Genomique Fonctionnelle de Lyon, CNRS UMR 5242, Ecole Normale Supérieure de Lyon, Université Claude Bernard, Lyon, France

**Keywords:** circadian rhythm, clock, genome topology, 3D genome, chromatin, DNA damage

## Abstract

Circadian rhythms orchestrate organismal physiology and behavior in order to anticipate daily changes in the environment. Virtually all cells have an internal rhythm that is synchronized every day by Zeitgebers (environmental cues). The synchrony between clocks within the animal enables the fitness and the health of organisms. Conversely, disruption of rhythms is linked to a variety of disorders: aging, cancer, metabolic diseases, and psychological disorders among others. At the cellular level, mammalian circadian rhythms are built on several layers of complexity. The transcriptional-translational feedback loop (TTFL) was the first to be described in the 90s. Thereafter oscillations in epigenetic marks highlighted the role of chromatin state in organizing the TTFL. More recently, studies on the 3D organization of the genome suggest that genome topology could be yet another layer of control on cellular circadian rhythms. The dynamic nature of genome topology over a solar day implies that the 3D mammalian genome has to be considered in the fourth dimension-in time. Whether oscillations in genome topology are a consequence of 24 h gene-expression or a driver of transcriptional cycles remains an open question. All said and done, circadian clock-gated phenomena such as gene expression, DNA damage response, cell metabolism and animal behavior—go hand in hand with 24 h rhythms in genome topology.

## Introduction

Our environment displays multiple cycles with different periodicities, from ultradian (period less than 24 h, as for tides), circadian (around 24 h, as for a solar day), and infradian (period greater than 24 h, as for seasons). Oscillations in light/dark cycles, availability of food and temperature rhythms are some of the most common circadian Zeitgebers (time-giver) encountered by organisms. Hence, virtually all species have evolved a circadian clock to adapt and anticipate such changes in environmental conditions. The presence of a circadian rhythm is defined on the basis of three criteria i) free-running period of ∼24-h, ii) ability to entrain to environmental cues, and iii) rhythms that are temperature compensated.

Clocks in different organisms seem to be the result of convergent evolution as the molecular core-clock components have diverged across the kingdoms of life. Circadian rhythms appeared in an ancestor of present-day cyanobacteria where the oscillator is built atop a post-translational phosphorylation-dephosphorylation cycle of the Kai A-B-C complex (Ishiura et al., 1998). It appeared independently a second time around in an ancestor of animals as a transcriptional-translational feedback loop (TTFL) (Dunlap, 1999; Rosbash, 2009). The clock allows anticipation of environmental conditions, adapting physiology, behavior, and metabolism to gate biological functions to specific hours along the day. In mammals, the hypothalamic suprachiasmatic nucleus acts as a master pacemaker to synchronize or entrain peripheral clocks distributed throughout the body. Within cells, the molecular mechanism of the core clock involves several layers of complexity orchestrated by transcriptional activators and repressors and chromatin state remodeling factors. More recently, with the establishment of powerful chromatin conformation capture methodologies, the importance of chromatin topology in the process has been highlighted. Altogether, cells and tissues generate multi-scaled rhythmic outputs from expression of gene networks to cell metabolism and global chromatin topology reorganization. These pathways then go on to regulate programs of DNA damage repair, cell cycle progression, physiology, and behavior.

The idea that DNA organization in the nucleus is non-random, exists for centuries (Rabl, 1885; Boveri, 1909). However, it is only in the 1980s that the theory of chromosome territories was validated by the development of Fluorescence *In Situ* Hybridization (FISH) (Manuelidis, 1985; Schardin et al., 1985). This technique gave crucial information about the spatial organization of the genome. Gene-rich chromosomes were found to localize in the center of the nucleus surrounded by gene-poor chromosomes, active-gene-rich regions were found to be in the core whereas inactive-genes regions were found to reside at the nuclear periphery in lamina-associated domains (LAD). Finally, it is only over the past 2 decades, with the completion of human genome sequence and the development of conformation detection techniques that nuclear architecture has shown a resurgence of interest. The initial breakthrough was the development of methods based on principles of ligation of linear distal genomic regions that come into close spatial proximity in 3D space, which allowed one to map genome interactions (Hakim and Misteli, 2012). The first technique developed was chromosome conformation capture (3C), a nuclear ligation assay in conjunction with qPCR, which allowed assessment of the proximity of two genomic loci (Dekker et al., 2002). Then with the development of third generation sequencing, protocols evolved to determine interactions between a single gene locus and the genome (4C) (Simonis et al., 2006), multiple loci against the full genome (Capture Hi-C) (Jäger et al., 2015), and finally at the level of the whole genome (Hi-C) (Lieberman-Aiden et al., 2009). These techniques have revealed the existence of chromatin domains 100 kb–1 Mb in size and regions displaying enriched interactions were named Topologically Associated Domains (TAD). These insulated domains are subdivided into smaller chromatin domains (“sub-TAD”). TADs are quite homogeneously active/euchromatin or inactive/heterochromatin and are not just structural but also functional and thus could act as a unit of gene regulation (Cavalli and Misteli, 2013; Dekker and Heard, 2015; Sexton and Cavalli, 2015). The precise principles governing TAD formation have not been elucidated yet. A proposed mechanism is chromatin loop extrusion that can be mediated by cohesin complexes and CCCTC-binding factor (CTCF) bound to its target motif. This favors the generation of promoter-enhancer or transcription-starting site (TSS)—transcription-terminating site (TTS) loops and genes found within a TAD often end up being co-regulated (Dekker and Heard, 2015; Le Dily and Beato, 2015).

This leap in technology gives a good overview of the genome organization in the nucleus and allowed one to ask if topological features are static or dynamic over time. Recent studies have focused on the genome topology reorganization under particular biological conditions such as during development, in aging, after an external cell stimulus, or in disease. A growing body of evidence now suggests that topological genome organization is rhythmic over 24 h-i.e. it cycles back to its original state at the start of every day. Thus, not only could this phenomenon be central in establishing circadian cycles in gene expression but also could be used in the daily control of DNA damage protection and/or repair pathways or regulation of aging over animal lifetimes.

In this review, we will focus on the core-mammalian clock mechanisms, followed by a discussion on the current evidence on how genome topology could be integrated into the mechanism of the core circadian clock. We will end by discussing the consequences of cycling genome topology on cellular functions.

## The Multiple Layers of the Circadian Core Clock

### The Transcriptional-Translational Feedback Loop

**FIGURE 1:**
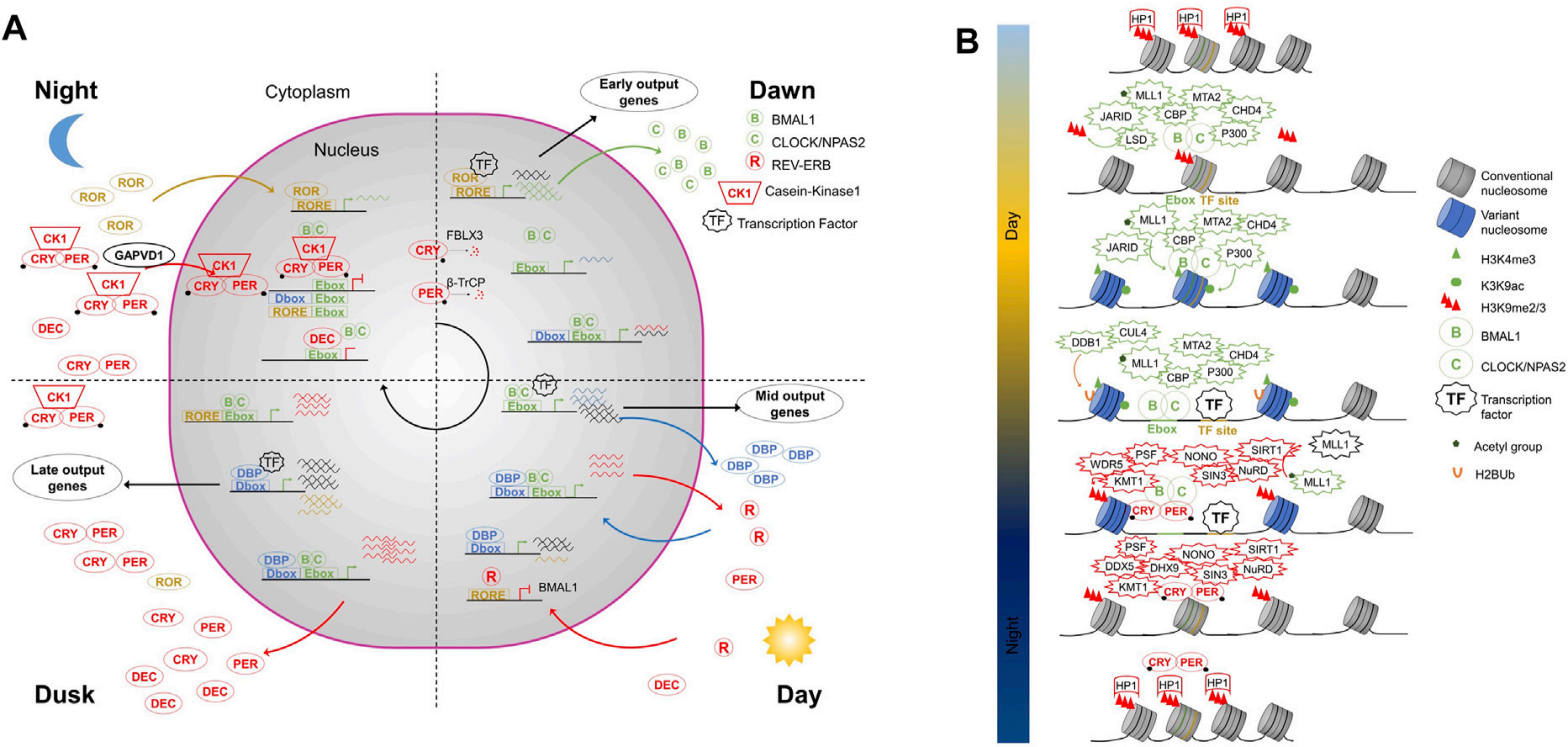
**(A)** The core circadian transcriptional feedback loop in mammals. Three interlocked transcriptional feedback loops build the core clock network. All three are based on the BMAL1-CLOCK transcription factor heterodimer that recognizes E-Box DNA sequence elements. i. BMAL1-CLOCK activates *Per* and *Cry* transcription whose protein products interact and inhibit their own transcription. The stability of PER and CRY is under the control of the E3-ubiquitin ligase β-TrCP and FBXL3, respectively. ii. BMAL1-CLOCK complex induces the nuclear receptors REV-ERB, which rhythmically repress BMAL1 expression driven by retinoic acid-related orphan receptor (ROR). iii. BMAL1-CLOCK complex induces DEC expression that in turn binds to E-Box elements in an competitive inhibitory manner. In addition to the feedback loops, the BMAL1-CLOCK complex induces the expression of the transcription factor DBP. Together, BMAL1-CLOCK, DBP, and ROR, in combination with cell and tissues specific transcription factors, allow the precise expression of circadian output genes at the right time. **(B)** Mammalian circadian chromatin. BMAL1-CLOCK complex can recognize E-box sequences engaged within nucleosomes. BMAL1-CLOCK recruits several epigenetic factors to alter chromatin state at clock genes. Consequently, and with the recruitment of other transcription factors, it activates transcription. At the end of the active phase, the PER-CRY complex binds to BMAL1-CLOCK and recruits epigenetic repressors. The megadalton complex formed establishes repressive chromatin states to inhibit transcription.

The mammalian clock is built on an evolutionarily conserved negative TTFL (Figure 1A) which is extensively described in excellent reviews elsewhere (Panda et al., 2002; Takahashi, 2017; Rosensweig and Green, 2020). The activators CLOCK (and its paralog NPAS2) and BMAL1, belonging to the basic helix-loop-helix-PER ARNT-SIM (bHLH-PAS) transcription factor family (Ledent et al., 2002), form a heterodimer to bind DNA regulatory elements containing E-boxes (CACGTG) and prime or activate transcription in mice tissues (Gekakis et al., 1998). Direct targets of BMAL1-CLOCK are the core clock repressors Period (PER1, PER2, and PER3) and Cryptochrome (CRY1 and CRY2) (Gekakis et al., 1998). In mice, the repressors start to be expressed in the afternoon and peak at the end of the day (Menet et al., 2012). PER and CRY proteins accumulate in the cytoplasm where they form a MegaDalton complex which includes the serine/threonine kinases casein kinase 1δ (CK1 δ) and CK1ε (Aryal et al., 2017). GAPVD1 has been proposed to chaperone the maturation and transport of the PER-CRY-CK1 complex to the nucleus (Aryal et al., 2017). Once in the nucleus PER-CRY complexes interact with BMAL1-CLOCK to inhibit transcription including their own expression (Lee et al., 2001). PER complexes contain helicases (DDX5, DHX9, and SETX) that are recruited to block transcriptional termination and cause a build-up of RNAP II (Padmanabhan et al., 2012). PER complexes also include NONO, a DNA- and RNA-binding protein. NONO depletion affects cellular rhythms (Brown et al., 2005) and could link the circadian clock with metabolic cues (Benegiamo et al., 2018) and the cell cycle (Kowalska et al., 2013). PER and CRY have a short half-life due to their degradation by the SCF E3 ubiquitin ligases β-TrCP (Shirogane et al., 2005) and FBXL (FBXL3 and FBXL21) (Busino et al., 2007; Godinho et al., 2007; Hirano et al., 2013; Yoo et al., 2013), respectively. Nighttime degradation in combination with the abrogation of their transcription leads to the rapid drop of PER and CRY concentrations in the morning (Wang et al., 2018). BMAL1-CLOCK transcription can start anew for a new cycle. In addition to PER-CRY, other complexes are also recruited to BMAL1-CLOCK to inhibit its activity as RACK1-PKCα that phosphorylates BMAL1 (Robles et al., 2010) or CIPC (Zhao et al., 2007). A second TTFL reinforces the core clock. Early during the day, BMAL1-CLOCK complexes induce REV-ERBα, REV-ERBβ, and DBP. DBP induces in turn retinoic acid-related orphan receptor α (RORα), RORβ, and RORγ at dusk and during the night (Yang et al., 2006). REV-ERB and RORs compete at ROR binding elements (RORE) allowing a cyclic transcription of BMAL1 and CLOCK, in antiphase to PER (Preitner et al., 2002; Sato et al., 2004; Zhang et al., 2015). A third TTFL, less studied, involves the Basic helix-loop-helix member E40 and E41 (also named DEC1 and DEC2). BMAL1-CLOCK induces DEC1 and DEC2 with a similar timing compared to PER and CRY. Subsequently, either DEC1 or DEC2 can inhibit the activity of BMAL1/CLOCK by directly binding to E-box elements (Honma et al., 2002). The transcription factors involved in the three TTFLs, i. e BMAL1-CLOCK, DBP, and ROR, have different activity time windows. Alone or in association with cell and tissue-specific transcription factors, they drive waves of gene expression at different times of the day in sync with the animal’s physiology (Trott and Menet, 2018).

### Circadian Chromatin Dynamics

The cyclic activation-repression of gene expression by the core clock transcription factor machinery relies on the secondary structure of the genome-the chromatin landscape. Genome compaction is achieved by the packaging of DNA into a repeated array of nucleosomes composed of histone octamers containing two molecules each of histones H2A, H2B, H3, and H4 (canonical histones) or their variants (e.g., H2A.Z) (Luger et al., 2012). The packaging of DNA in chromatin is not static and serves as a basis for gene regulation. Post-translational modification of nucleosomes, the variant composition of nucleosomes, and positioning/spacing of nucleosomes together constitute an enigmatic epigenetic code that regulates gene expression (Allis and Jenuwein, 2016). Circadian oscillations in all these three major pathways have been linked to the function of the core clock and its outputs (Figure 1B) (Koike et al., 2012; Le Martelot et al., 2012).

During the activation phase, the BMAL1-CLOCK complex has been shown to recruit lysine-specific histone demethylase JARID1A and LSD1 that could remove the repressive H3 lys9 di- and tri-methyl (H3K9me2/3) marks (DiTacchio et al., 2011; Nam et al., 2014). The disappearance of H3K9me2/3 releases HP1 and thus favors chromatin de-condensation. Moreover, CLOCK is thought to have histone acetyltransferase activity (HAT); it positively imprints the chromatin by acetylating H3K9 and H3K14 (Doi et al., 2006). BMAL1-CLOCK brings P300 and CREB-binding protein (CBP), other HATs, which could increase, accelerate or reinforce the robustness of histone acetylation (Etchegaray et al., 2003; Hosoda et al., 2009). In parallel, the complex brings acetylated MLL1 to methylate H3K4 (Katada and Sassone-Corsi, 2010), an activity that slows down when SIRT1 is recruited. SIRT1 inactivates MLL1 by deacetylating it (Aguilar-Arnal et al., 2015).

To prepare the repressive phase, the BMAL1-CLOCK complexes mark nucleosomes with ubiquitin moieties at their target sites with the aid of DDB1-CUL4 complexes. The resulting mono-ubiquitinated H2B nucleosomes participates in PER complex recruitment (Tamayo et al., 2015). The repressive PER complexes contain PSF that acts as a scaffold for recruitment of repressor complexes SIN3-HDAC (Duong et al., 2011) and HP1γ-SUV39H (Duong and Weitz, 2014), which deacetylates histones H3K9, and H4K5 and di-tri-methylate H3K9, respectively. The methyltransferase WDR5 also interacts with the PER complex and actively participates in the repressive methylation cycles (Brown et al., 2005). CHRONO, a recently discovered clock component interacts with BMAL1-CLOCK independently of the PER complex to repress transcription in a deacetylation-dependent manner (Anafi et al., 2014; Goriki et al., 2014; Yang et al., 2020). The deacetylation, methylation, and recruitment of HP1 in turn, changes the nature of chromatin, allowing it to condense to form facultative heterochromatin and inhibit transcription.

In addition to post-translational modifications, canonical histones can also be exchanged for histone variants. The variant histones have specific characteristics compared to canonical counterparts. In addition to not having the same residues for post-translational modification they also have varying affinity of interactions with other histones, DNA and act as a scaffold for chromatin interactor recruitment. The histone variant H2A.Z has been proposed to participate in chromatin opening due to a higher density of these nucleosomes around BMAL1 binding site following TF binding (Menet et al., 2014) but functional studies are lacking. The histone variant MacroH2A1 has been shown to impact circadian rhythms in a human hepatocarcinoma cell line model by regulating core-clock gene expression (Carbone et al., 2021).

Finally, remodeling of nucleosome array can also participate in the activation and repression of circadian gene expression. At the beginning of the activation phase, the BMAL1-CLOCK complex, which is thought to act as a pioneer factor, recognizes E-box sequences engaged within nucleosomes in facultative heterochromatin and favors nucleosome eviction creating nucleosome depleted regions (Menet et al., 2014). Associated with the activator complex are components of the Nucleosome Remodeling and deacetylase (NuRD) complex, CHD4, and MTA2 (Kim et al., 2014). Despite the well-described role of the NuRD complex in repression, some activator functions have also been described (Hoffmeister et al., 2017). In the circadian context, CHD4 actively participates in the activation of transcription (Kim et al., 2014) likely due to its chromatin remodeling action and in association with additional nucleosome remodeling factors such as SNF2h or BRG1. MTA2, which is also found in the BMAL1-CLOCK complex, acts purely in feedback transcriptional repression (Kim et al., 2014) and could be important for the proper assembly of the full NuRD complex (Zhang et al., 1999). At the end of the active phase, the PER complex brings the other “half” of the NuRD complex, MBD2, GATAD2a, and HDAC1 that are required for the repressive action of the NuRD complex and thus imparts specificity to the clock (Kim et al., 2014).

Experiments to determine the full repertoire of chromatin modifiers and remodelers that are at the core of the circadian oscillator are still ongoing. The mechanism by which cells coordinate gene-specific remodelers at the genome-scale is likely linked to the higher layer of gene expression control - at the level of 3D organization of the genome.

### The Spatio-Temporal Organization of Circadian Chromatin

Starting from a linear fiber of DNA to the highly condensed mitotic chromosome, there exists several intermediate levels of chromatin organization. Chromatin forms loops which can be grouped into TADs and sub-TADs are split between compartment A (transcriptionally active) and compartment B (transcriptionally repressed) (Dixon et al., 2012). TADs can be recruited to the lamina generating Lamina Associated Domains (LADs) that are heterochromatic by nature (Gonzalez-Sandoval and Gasser, 2016). In addition to this primary layer of chromatin organization, each chromosome is associated with an allocated territory within the nucleus (Krumm and Duan, 2019). In general, chromosome territories and TADs are well-conserved among tissues and even likely among species (Dixon et al., 2012; Nora et al., 2012; Harmston et al., 2017; Eres and Gilad, 2021).

A series of studies have explored the cause and consequences of genome topology on the control of gene expression in general but also more specifically within the circadian context (for previous reviews on this topic, we refer you to (Yeung and Naef, 2018; Pacheco-Bernal et al., 2019)). The first evidence that genome topology takes part in the circadian mechanism came from chromosome conformation capture on chip (4C) experiments (Aguilar-Arnal et al., 2013). In mouse embryonic fibroblasts, using the *Dbp* locus as a viewpoint, interactions with 200 genomic interchromosomal loci were observed in all, of which 29 were oscillating. Knocking out *Bmal1* in these cells specifically disrupted the oscillating interactions revealing the need for the core clock in the dynamic *Dbp* gene interactome. Investigations at the *Nr1d1* locus (coding REV-ERBα) revealed stable interactions during the circadian cycle that are important for the cyclic activation of transcription (Xu et al., 2016). Cohesin, independently of CTCF, is particularly important in chromatin topology as it allows the formation of a hub of co-regulated genes by bringing together enhancers and promoters. In the murine liver, cohesin in association with CTCF allows the insulation of specific domains of the chromatin, and was shown to separate circadian TADs according to the acrophase, and circadian TADs from non-circadian TADs (Xu et al., 2016).

**FIGURE 2:**
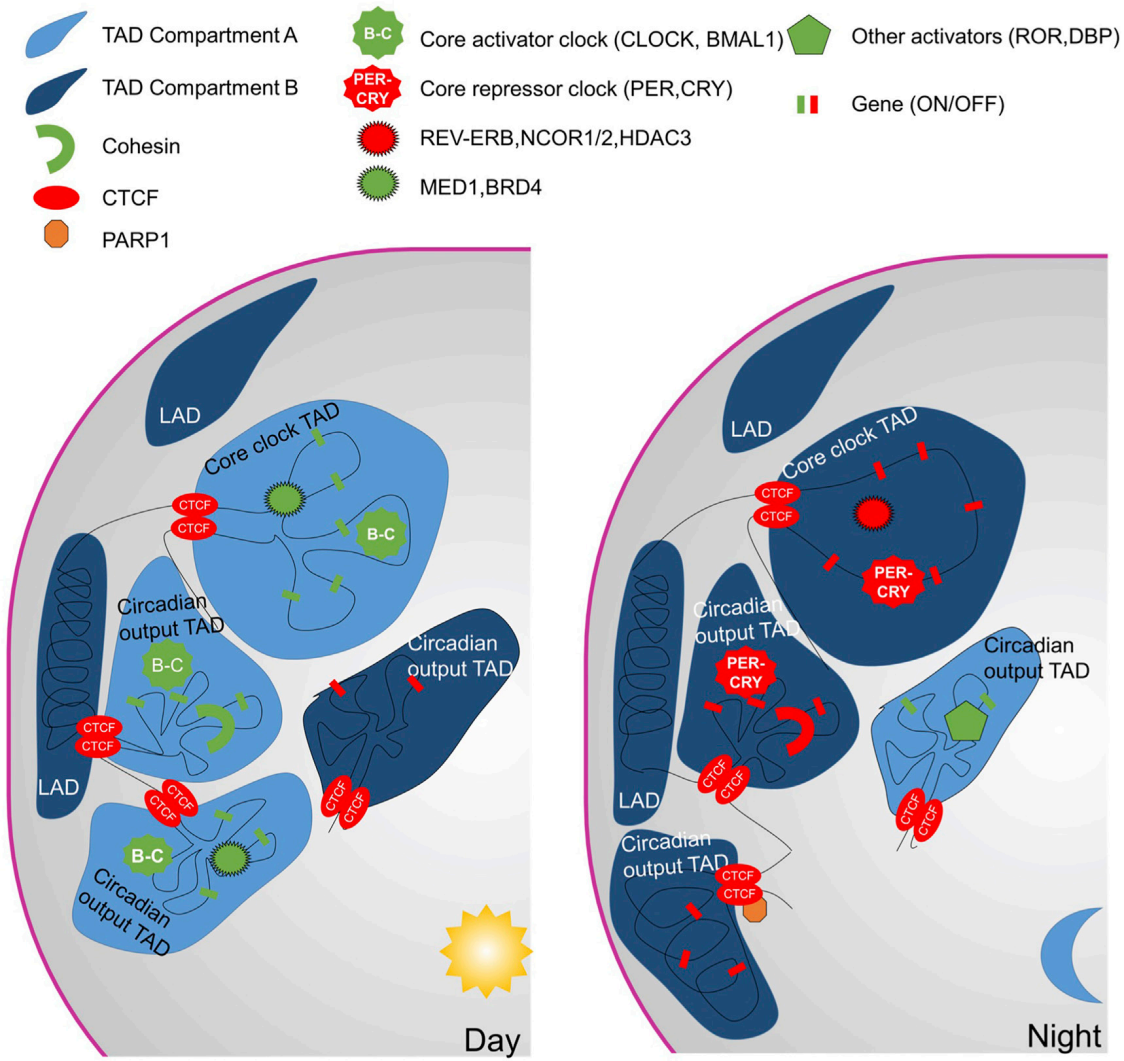
Spatio-temporal regulation of chromatin. During the active phase, most of the circadian TADs are in compartment A (euchromatin) with numerous intra-TADs interactions. Cohesin, Med1 and BRD4 participate in the intra-TAD interactions. The TADs form hubs for co-transcriptional regulation by BMAL1-CLOCK. In addition, for the core-clock genes, enhancer-promotor loops activate gene transcription. The shift to the repressive phase is accompanied by a transition of a circadian TAD from compartment A to B. The intra-TAD interactions are maintained for clock output genes whereas promotor-enhancer loops engaged for core-clock genes dissociate.

Furthermore, the Lazar group deployed the Hi-C method to define interactions between two genomic loci (Kim et al., 2018) at two diurnal time points. Around 900 “intra-TAD” oscillating interactions were revealed whereas TAD borders were observed to remain stable. The importance of REV-ERBα was highlighted in establishing these domains. REV-ERBα abrogates chromatin interactions to inhibit transcription during the day in mice (Figure 2). To do so, REV-ERBα recruits NCoR-HDAC3 and removes the elongation factor BRD4 and the looping factor MED1. Nevertheless, not all REV-ERBα binding sites are equivalent and at “passive” sites REV-ERBα recruitment had no effect on loop dissociation. Despite the demonstrated role of REV-ERBα in circadian genome topology, the function of REV-ERBα seems to be mainly on the regulation of output genes as cells depleted of REV-ERBα and REV-ERBβ still have a functional core-clock (Ikeda et al., 2019).

To better understand the importance of the chromatin topology on a role in the function of the core clock or the expression of the circadian output genes, two other studies focused on the circadian chromatin topology (Furlan-Magaril et al., 2021; Mermet et al., 2021). The Naef group used 4C-seq to follow the interactions of several loci with the rest of the genome over 24 h (Mermet et al., 2021). Three promoters of important core clock genes, *Bmal1 Per1* and *Per2* were shown to be involved in oscillating interactions with surrounding enhancers. The time of the highest interactions between promoter and enhancers precedes pre-mRNA synthesis. To determine if oscillating genomic contacts are specific to the core clock or general among oscillating genes, several output gene loci were also considered (*Mreg*, *Nampt*, *Pfkfb3*, *Msfd2a*, and *Por*). Most of these loci showed stable interactions with enhancers over time and importantly these were observed also in arrhythmic *Bmal1* ^*−/−*^ animals. Output genes seem to arrange in a chromatin hub of temporally co-transcribed genes. An independent study from the Fraser group arrived at a similar conclusion but at a genome-wide scale (Furlan-Magaril et al., 2021). To study chromatin contacts of promoters at high resolution over time, they applied promoter capture Hi-C. The analysis revealed that core clock loci tend to contact fewer genomic elements but are more dynamic. Contrarily, output genes revealed more interactions which were also more stable. The genome-scale analysis shows that TAD borders are stable over time and circadian genes with a shared transcriptional phase are grouped within a TAD. In addition, the chromatin within a circadian TAD oscillated between the A and B compartments (from active to inactive) over the course of a day. Reanalysis of the REV-ERBα HiC experiment (Kim et al., 2018) considering not just two but three categories: REV-ERBα engaged sites associated with core-clock genes, REV-ERBα engaged sites associated with clock-output genes and REV-ERBα passive sites, could also prove interesting in light of these recent observations.

In sum, clock output genes tend to form hubs that allow co-expression of genes with the same acrophase (Figure 2). While numerous chromatin contacts are formed between promoters and enhancers at both core-clock and output genes, the majority of the interactions stay stable over the day for the outputs. Contrarily, core clock genes tend to be controlled by few specific dynamic contacts with genomic regulatory elements. The distinction, at the gene topological level, highlighted between core-clock genes and clock-output genes (Furlan-Magaril et al., 2021; Mermet et al., 2021) is unexpected and significant and raises important questions about the how, why and for what. The highly dynamic interactome, specific to core clock genes, suggests another layer of control of BMAL1:CLOCK mediated circadian gene expression that ensures in its robustness whereas the TADs could be the unit of regulation of output genes. Mechanisms underlying these different behaviors are waiting to be discovered.

Some clues could come from the nuclear localization of chromatin domains in addition to chromatin folding. For example, at the nuclear periphery, chromatin associated with the membrane form lamina-associated domains (LAD). LADs are typically heterochromatic in nature. In 2015, Zhao et al. demonstrated that PARP1 targets CTCF-bound chromatin to LADs to inhibit circadian gene expression (Zhao et al., 2015). The experiments were performed on embryonic stem cells or embryonic bodies that do not have robust clocks (Zhao et al., 2015; Benitah and Welz, 2020) and confirmed on HCT116 cancerous cells (Zhao et al., 2015). In addition, it is interesting to note that DEC1 can interact with CTCF and reinforce chromatin loops formed by CTCF (Hu et al., 2020). Since DEC1 can bind E-boxes, in coordination with a CTCF-PARP1 complex it could, in theory, target chromatin to LADs for transcriptional inhibition. Nevertheless, more work *in vivo* will be required to confirm these results obtained from cultured cells, especially since a study from the Collas lab showed that chromatin interactions with nuclear lamina are uncoupled from rhythmic gene expression in synchronized liver tissue (Brunet et al., 2019).

Additional work on the nuclear location of core-clock and output gene loci will be required to understand the roles of the interactions. Are genomic interactions by themselves the crucial topological features of the core clock and output gene expression? Or do genomic interactions participate in relocation of the loci in 3D space required for gene repression? It is tempting to speculate that because core clock genes form transient loops during the day, the enhancer-promoter interactions are directly involved in the regulation. Could the hub could move from euchromatin territories to heterochromatin territories like the lamina or around the nucleolus? The requirement of chromatin topology modifications to build the circadian core clock is fertile for the next phase of investigations.

## Consequences of Genome Topology Oscillations Beyond Gene Expression

The circadian clock integrates several layers of regulation from the activator-repressor complexes to genome topological oscillations via chromatin state modifications. Such a robustly controlled self-sustained mechanism not only manages the expression of output genes but also could use these dynamics for other functions within cells. One such phenomenon in differentiated cells is the DNA damage and repair response.

Genomic integrity is at constant risk of damage from solar ultraviolet radiation, from metabolism-derived reactive species and xenobiotics. The risk to the genome from such phenomena are spread out over 24 h and is linked to light exposure that affects exposed organs such as the skin, while activity and feeding cycles typically impact visceral tissues. A description of DNA damage pathways and damage response and repair (DDRR) is beyond the topic of this review, and we invite readers to other excellent reviews on this topic (Sirbu and Cortez, 2013). DDRR pathways, which include DNA repair, cell cycle arrest or death have evolved to limit genomic damages that happen during the day. Human keratinocytes activate pathways that offer protection from UV in anticipation of the incoming radiation-induced damage every day (Janich et al., 2013) Moreover, the sensitivity to rhythmic damage is lost in the absence of core clock components such as BMAL1 or the CRYs (Geyfman et al., 2012). While circadian machinery may be expendable for DDRR to work (Gaddameedhi et al., 2012), some genes of the DDRR such as XPA were shown to be cyclically expressed and the activity of the DDRR pathways such as nucleotide excision repair was shown to be cyclic (Gaddameedhi et al., 2012; Geyfman et al., 2012; Yang et al., 2018).

It has notably been shown that condensed heterochromatin is more resistant to DNA damage and protected from double-strand breaks (DSB) while more open chromatin is more sensitive to such damage (Cann and Dellaire, 2011; Takata et al., 2013; Nair et al., 2017). One of the reasons is that DNA damaging agents have less likelihood of affecting genomic sites protected either by local chromatin architecture or protein complexes when compared to open/accessible DNA. Nevertheless, at the same time, the DDRR machinery needs access to the damaged loci to repair the damage. DDRR therefore is more active in open chromatin (Adar et al., 2016; Nair et al., 2017). Finally, even if heterochromatin is less prone to DNA damage, the lower accessibility for DDRR factors induces higher rates of mutagenesis in heterochromatin (Schuster-Böckler and Lehner, 2012; Zheng et al., 2014; Mao and Wyrick, 2019).

Interestingly, in mice, a non-negligible part of chromatin tends to be more open during the day and more condensed during the night (chromatin marks (Koike et al., 2012), DNAse sensitivity (Sobel et al., 2017), compartment repartition (Furlan-Magaril et al., 2021)). Bee et al. showed that in clock-synchronized cell lines micrococcal nuclease accessibility as well as chromatin condensation measured by fluorochrome binding differed in two dishes of cells 12 h antiphase to each other (Hood and Amir, 2017). Moreover, cells are more prone to DNA damage and better repair during the time of the day with high BMAL1 protein content which correlated with relaxed chromatin (Bee et al., 2015). While these experiments do not approach the resolution provided by chromatin-capture technologies, it would be interesting to determine if DNA damage and DNA repair pathways correlate with oscillating and non-oscillating TADs that move from compartment A to B in a circadian manner i.e. the crosstalk between circadian chromatin topology and circadian DNA damage and DDRR.

## Conclusion and Perspectives

The circadian clock uses numerous mechanisms to sustain robust and precise oscillations. Over 3 decades of work have identified core-clock components and the competition between activators (BMAL1, CLOCK, and ROR) and repressors (PER, CRY, REV-ERB, and DEC) that build the loop. The discovery of chromatin organization dynamics followed after. The clock complexes recruit multiple chromatin modifiers and so epigenetic marks oscillate over 24 h. Finally, it is only recently that genome topology dynamics have been implicated in the core clock mechanism with specific features that distinguish it from the outputs of the clock. The core clock loci form few and dynamic contacts whereas output loci make numerous but stable interactions. Nevertheless, work is still required to understand the requirement of genome topology in the circadian clock and if the folding of the chromatin is sufficient or if the spatial displacement of circadian regions in nuclear space is also a prerequisite. Whether pathways that are identified in the liver or cell lines apply to all tissues/cells where peripheral clocks function also remains open to study.

How does the aging clock alter chromatin and genome topology? During aging, rhythms tend to weaken with damped amplitudes and shorter periods (Chen et al., 2016; Hood and Amir, 2017) and at its extreme, clock defective mice models (especially *Bmal1* knockouts) age extremely rapidly (Kondratov et al., 2006). Cells from aged individuals often exhibit reduced areas of heterochromatin, loss of repressive histone marks, the altered composition of core histones and histone variants, and appearance of nucleosome-depleted regions (Cavalli and Misteli, 2013). Nevertheless, rhythms in tissues from luciferase mouse models can stay remarkably stable over time (Canaple et al., 2018). Are core-clock loci and the topological domains that house them immune to the aging effect? Following increasing evidence for reciprocal interactions between aging and circadian rhythms (Kondratova and Kondratov, 2012), a focus on chromatin organization and genome topology could hold the key to decrypting the underlying mechanism. This will help design bi-directional therapies: regimens that stabilize the circadian clock would favor healthy aging, while improving healthspan would strengthen the clock and control of animal physiology, metabolism and behavior (Eckel-Mahan and Sassone-Corsi, 2013; Akashi et al., 2020).
